# Outcome of Surgical Treatment of Pelvic Osteosarcoma: Hospital Universiti Sains Malaysia Experience

**DOI:** 10.5704/MOJ.1303.018

**Published:** 2013-03

**Authors:** MS Ariff, W Zulmi, WI Faisham, MZ Nor Azman, AH Nawaz

**Affiliations:** Department of Orthopaedics, Traumatology and Rehabilitation,International Islamic University Malaysia, Kuantan, Malaysia; Department of Orthopaedics, Universiti Sains Malaysia, Kubang Kerian, Kelantan; Department of Orthopaedics, Universiti Sains Malaysia, Kubang Kerian, Kelantan; Department of Orthopaedics, Universiti Sains Malaysia, Kubang Kerian, Kelantan; Department of Orthopaedics, Universiti Sains Malaysia, Kubang Kerian, Kelantan

## Abstract

We reviewed the surgical treatment and outcomes of 13 patients with pelvic osteosarcoma treated in our centre in the past decade. The study sample included 9 males and 4 females with a mean age of 28.1 years. Four patients had ileal lesions, five had acetabulum lesions, one had a ischiopubis lesion, and three had involvement of the whole hemipelvis. Seven patients presented with distant metastases at diagnosis. Limb salvage was performed in 6 patients and amputation in 7. In 60% of cases in the limb salvage surgery group, we attempted wide resection with positive microscopic margin compared to only 16.7% in the amputation group. Local recurrence was higher in the limb salvage group. Overall survival was 18 months for mean follow up of 14.8 months. Median survival was 19 months in the limb salvage group compared to 9 months in amputation group. The outcome of surgical treatment of pelvic osteosarcoma remains poor despite advancements in musculoskeletal oncology treatment.

**Key Words:**

Pelvic osteosarcoma, limb salvage surgery, hemipelvectomy, margins, oncologic outcomes

## Introduction

Pelvic osteosarcomas are rare, accounting for less than 10% of all osteosarcomas^1^-^4^. Like other pelvic tumours, patients with pelvic osteosarcoma typically present at later stages with large tumour size; pulmonary metastases at the time of diagnosis are not uncommon. These tumours are in close proximity to the pelvic organs and neurovascular structures, and poor compartmentalization in the pelvis complicates attempts to achieve local control through adequate surgical margins^1^,^4^-^6^. Histologically, pelvic osteosarcomas are usually chondroblastic in nature; similiar to pelvic chondrosarcoma, such tumours respond poorly to chemotherapy and radiotherapy^1^,^2^,^5^-^7^. Hence, surgical management of pelvic osteosarcoma is quite challenging and continues to be associated with significant mortality and morbidiy despite advancement in multimodality treatment of musculoskeletal tumours.

Only a few large series of pelvic osteosarcomas surgical outcomes are available in the literature. Overall survival rates for pelvic osteosarcoma patients range from 20% to 50%, far worse than reported survival rates for osteosarcoma of the limbs^5^-^10^. Since most reports are from single institutions, it is unclear whether the reported rates are universal for all pelvic osteosarcoma patients or specific to the respective institutions^3^.

For almost two decades, in the Orthopaedic Oncology and Reconstruction Unit of Hospital Universiti Sains Malaysia, pelvic osteosarcomas have been treated with a multidisciplinary approach. We would like to review the treatment outcome of this condition.

## Materials and Methods

Thirteen patients with osteosarcoma of the pelvis were treated surgically from January 2001 to December 2010. Clinical and radiological records of all patients were reviewed. Routine haematological and biochemical investigation, magnetic resonance imaging (MRI) of the primary tumour, whole body technetium 99 bone scintigraphy and computed tomography (CT) of the chest were conducted as part of the staging workup. Tissue diagnoses were obtained in all cases with either open or trucut biopsy.

Anatomic sites and types of resection were classified into type I (ilium), type II (acetabulum) and type III (ischiopubis), or combinations thereof, based on the system established by Enneking and Dunham^11^. Hemipelvic type is assigned when the tumour involves all three regions. Osteosarcomas of the sacrum and sacroiliac region were excluded from this study unless a majority of the tumour was located in the ilium based on radiological reports. Tumour volume was calculated according to the prevously described methods^12^-^14^. Tumour volume of > 1000ml were defined as large tumours, 500 to 1000ml were defined as moderate and < 500ml were defined as small tumours. Tumours were staged according to the Musculoskeletal Tumor Society (MSTS) staging system developed by Enneking^15^.

Patients received neoadjuvant chemotherapy consisting of adriamycin and cisplatinum according to the European Osteosarcoma Intergroup (EOI) protocol. Neoadjuvant chemotherapy was not prescribed in cases with chondroblastic subtype and those that required urgent surgical intervention to avoid delay. In patients who received neoadjuvant chemotherapy, surgery was performed after two to four cycles of chemotherapy. Repeat MRI evaluation for staging before surgery to assess response to chemotherapy and extent of primary tumour was performed two weeks before surgery. Limb salvage surgery (LSS) was attempted in most cases, but the final decision was made at the time of surgery and based on the ability to completely resect the tumour en bloc with preservation of the neurovascular bundle. Otherwise, patients were subjected to external hemipelvectomy. Various types of external hemipelvectomy were used based on the level of resection needed^16^. A classical hemipelvectomy or hindquarter amputation involves removal of the whole hemipelvis through the sacroiliac joint and pubic symphysis, together with the ipsilateral lower limb. A modified hemipelvectomy preserves part of the iliac crest. An extended hemipelvectomy consists of the standard procedure along with surgical removal of contiguous musculoskeletal structures, such as the lumbar spine, contralateral pelvic bones, or sacral elements. A compound hemipelvectomy involves resection of visceral pelvic structures in addition to the affected pelvic bone.

Wide resections consisting of tumour removal en bloc with a cuff of normal tissue around the mass were attempted in all patients with curative intent. For palliative cases, marginal resection was performed. Surgical specimens were evaluated for microscopic extension at tumour margins. Postoperatively, all patients, except for one with chondroblastic osteosarcoma, completed chemotherapy with or without radiotherapy. Patients were then assessed every three to four month. Repeat radiological assessment was performed when warranted, based on symptoms. Serial CT scans of the chest and whole body bone scans were performed every six months. Actuarial survivals of patients were estimated using Kaplan-Meier’s survival plots.

## Results

There were nine males and four females with a mean age of 28.1 years (range, 8- 52 years). Tumours were located in the ilium/ iliosacrum in four cases, acetabular region in five cases and in the ischiopubis in one case. The whole ipsilateral hemipelvis was involved in three cases. A majority presented with extracompartmental involvement with soft tissue infiltration (Stage IIB Enneking classification). Five patients had pulmonary metastases at the time of presentation. Three patients, all in stage III, had previous surgical treatment in other institutions and presented with local recurrence. All cases in this series were high-grade osteosarcoma. Eight cases (61.5%) were osteoblastic osteosarcoma and other subtypes included chondroblastic osteosarcoma, spindle cell osteosarcoma, fibrohistiocytic variant, fibroblastic osteosarcoma, and poorly-differentiated osteosarcoma (one case (7.7%) of each subtype).

**Fig. 1:**
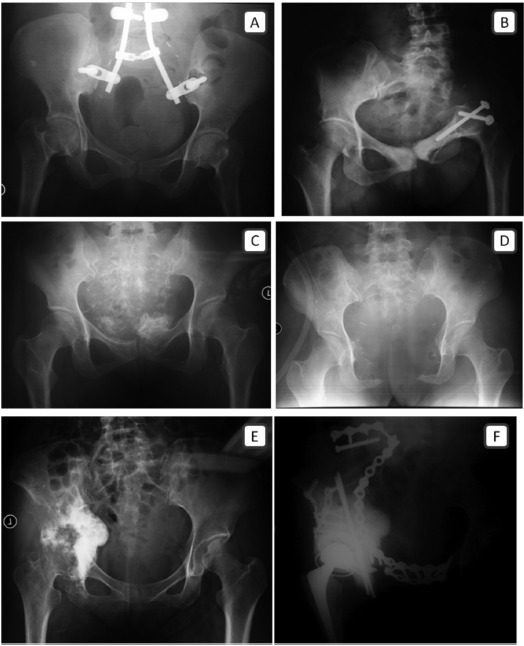
: Anteroposterior plain radiographs of the pelvis. Galveston iliolumbar fusion following iliosacral resection (A). Ischiofemoral arthrodesis following ilioacetabular resection (B). Osteosarcoma of the pubic bone resection without reconstruction (C, D). Osteosarcoma involving the ipsilateral ischiopubis, acetabulum and ilium, resected and reconstructed with a modified Harrington procedure (E, F).

Eight patients had large tumour volume, and the median volume was 1122.5ml (range, 19ml- 4500ml). Limb salvage surgery was performed in six patients with three cases of type I resection, two type II resections and a single case of type III resection. Following type I resection, two cases need no further reconstruction; only 1 case was reconstructed with Galveston iliolumbar fusion with instrumentation and allograft (Figure 1a). The single case of type III resection did not require any further reconstruction as the hip joint was not involved (Figure 1c and 1d). Two patients that underwent type II resection received different types of reconstruction. In the first case, ischiofemoral arthrodesis was performed (Figure 1b), and the second patient was subjected to extracorporeal radiation followed by reconstruction with a modified Harrington procedure (Figure 1e and 1f). External hemipelvectomy was necessary in seven cases, five were external hemipelvectomies, and three others were one each of modified and extended type.

Wide resection was performed in all cases with curative intention; five cases in the LSS group and six cases in the amputation group. Marginal resection was performed in one case of type I palliative resection and another case of palliative amputation. Three out of five cases from the LSS group (60.0%) with attempted wide resection had positive microscopic margins compared to only one out of six (16.7%) in the amputation group (Table I). Local oncologic clearance was better achieved in stage III tumours and tumours with large volume.

**Fig. 2:**
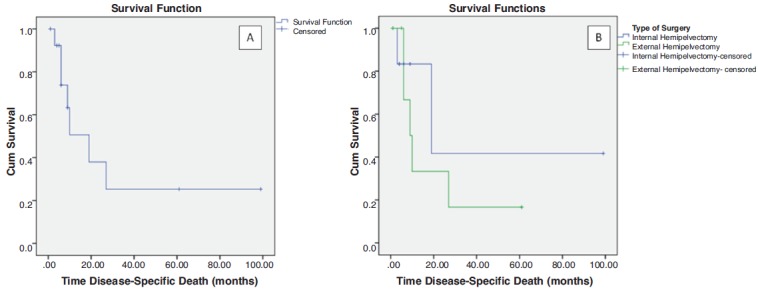
: Kaplan-Meier curve. Overall survival of the entire cohort (A), survival according to types of surgery (B).

Microscopic margins were negative in 7 patients; 4 cases of stage IIA and IIB were free of disease. Two cases of stage III survived with disease and 4 died of the disease. Six cases had positive microscopic margin; 4 cases of stage IIA and IIB survived without disease, one survived with disease and another died of the disease. Two cases of stage III survived with the disease at 12 months. Overall median survival time was 18 months for a mean follow up of 14.8 months (range 1-100 months). Median survival of the LSS group was 19 months compared to 9 months in the amputation group (Figure 2b). Only one amputee had local recurrence within the first post-surgery year compared to two cases from the LSS group. Both cases had positive microscopic margins.

The actual oncologic status of patients at the last follow-up in relation to stage of tumour, achieved surgical margin and microscopic margin, and the types of surgical treatment is summarized in Table II. Two out of five patients with stage IIB osteosarcoma underwent amputation. Both had no evidence of recurrence on last follow-up. The patient who underwent type II resection followed by modified Harrington reconstruction had local recurrence after eight months and subsequently underwent external hemipelvectomy. She survived for almost nine month after the second surgery. Seven patients had distant metastases at presentation, with two patients presenting with distant sites other than pulmonary metastases. A majority of patients (3 of 5) with pulmonary metastases at presentation died within one year after surgery. The only patient with distant metasteses who underwent LSS succumbed to the disease three months after surgery.

Two of four amputated patients survived with the disease for more than 1 year with the longest up to 31 months. The longest survivor was not responsive to chemotherapy and was diagnosed with secondary acute myeloid leukaemia (AML) two years after surgery. All amputees had local flap complications, including flap congestion, wound dehiscence and infection. A majority of patients (4 of 6) from the LSS group also had local wound complications.

Two LSS patients had neurological deficits after surgery. Complications seemed to be related to the types of surgery rather than age-related.

## Discussion

Although large series have been reported on pelvic osteosarcoma, information is still limited^3^,^4^,^6^,^7^,^17^-^19^ particularly for reported series from this global region. This makes comparison of the current series with others more difficult. Cases in the current series were slightly different than those previously reported. A majority of our cases were osteoblastic in nature and not chondroblastic. Therefore, most of our patients underwent neo-adjuvant chemotherapy. Classically, tumour size is used for staging and prognostication of tumours, and in fact, there is a trend now towards the use of tumour volume to predict the risk of metastases and prognosis^14^,^20^. As such, we also used tumour volume to predict prognosis and metasteses. The anatomical distribution of tumours in this study correlates with what was described by Enneking^21^, in which the most common site is the iliosacral region.

Pelvic osteosarcomas are difficult to treat, even with the advancement of multimodal oncologic services. Excellent response to chemotherapy in the treatment of osteosarcoma assist surgeons to achieve adequate local control. Unfortunately, in pelvic osteosarcoma, there is typically a poor response to chemotherapy^6^-^8^, ^22^,^23^. Large tumour size or volume further decrease response to chemotherapy while at the same time making it difficult to resect without marked morbidity^8^, ^23^-^24^. Furthermore, the median age is higher for pelvic osteosarcoma patients compared to patients with extremity osteosarcoma, and therefore a lower chemotherapy dose intensity^17^. Late presentation and large tumour mass is still a widespread problem among patients in this region especially since a large number of patients still opt for complementary-alternative medicine for orthopaedic problems before presenting to the medical center^25^.

Misdiagnosis and late detection of pelvic tumours is still relatively common even among experienced surgeons^26^-^29^. Since early 2000, osteosarcoma patients treated in our centre were subjected to chemotherapy according to European Osteosarcoma Intergroup (EOI) protocol. We use adriamycin and cisplatinum with additional ifosfamide if there is significant soft tissue involvement. Survival rates of patients were evaluated in a short review in 2004 that showed an overall survival rate of 52%, and survival rate of 78% in the limb salvage group^30^.

Randomized trials have demonstrated that the two-drug regimen has outcomes comparable to the multi-drug regimen incorporating methotrexate^31^ with a more complex and longer schedule^32^,^33^. In a recent study using the combination of adriamycin, cisplatinum, and ifosfomide, there was a significant association between good responses and compliant patients^34^. Hence, institutions with limited resources shoud endeavor to provide pharmacokinetic monitoring for methotrexate^33^,^35^.

As expected, large volume tumour resistance to chemotherapy is associated with higher risk of pulmonary metastases^20^-^29^, and both factors carry a very poor prognosis in pelvic osteosarcomas^12^-^13^,^22^-^32^. Another important prognostic factor is the adequacy of tumour resection^5^,^8^. Despite the growing trend towards LSS, previous reports showed amputation has a better rate of adequate surgical margins^7^. Similarly, a majority of LSS patients in the current series had positive margins compared to amputation group (Table II). Previously, the probability of achieving a clear microscopic margin in pelvic reseections with attempted wide resections was only 50%^36^. Similar findings were reported in previous series^5^,^17^. Thus, external hemipelvectomy should be selected in cases where LSS may compromise margin adequacy considering the high risk of local recurrence. Radiotherapy should be considered when adequate local control cannot be obtained from surgery. Due to the limited number of patients in the current series, we did not evaluate the association between anatomic site, tumour stage, and tumour volume with the incidence of positive microscopic margins following resection. Overall survival of patients in the current series is comparable to the lower end of rates reported in previous published series, between 20% and 50% 1,4-8,17.

**Table I:**
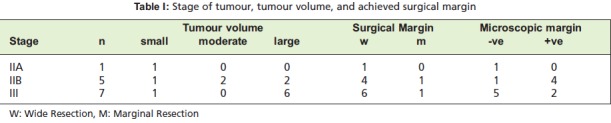
: Stage of tumour, tumour volume, and achieved surgical margin

**Table II:**
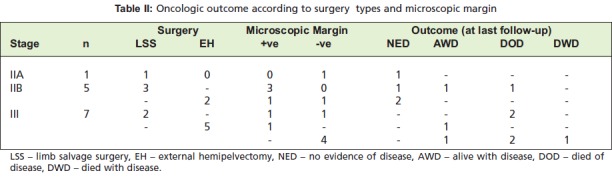
: Oncologic outcome according to surgery types and microscopic margin

## Conclusion

External hemipelvectomy provided fairly good local control but was associated with significant morbidity. Hemipelvectomy is viable as a curative procedure for locally advanced osteosarcoma of the pelvis. Survival was influenced by the presence of pulmonary metastases as well as the status of microscopic margin. Technical challenges, infrequent indications for these procedures, and anticipated complications demands that resection of these tumours be performed only in highly specialized tertiary medical institutions with a comprehensive, multidisciplinary, surgical oncology team, and state-of-the-art facilities.
